# Supplementary material for: Identification of photoperiod-induced specific miRNAs in the adrenal glands of Sunite sheep (Ovis aries)
Source: Front Vet Sci. 2022 Jul 22;9:888207. doi: 10.3389/fvets.2022.888207 (PMC9354845; doi:10.3389/fvets.2022.888207)
Supplement: Supplementary file 1 [file Data_Sheet_1.ZIP › Supplementary file/miRDeep2 file.zip!/miRDeep2 file/Novel_Structure/SP42A3/Novel_440.pdf]

Provisional ID	:	NC_019464.2_13234
Score total	:	1.2
Score for star read(s)	:	-1.3
Score for read counts	:	-0.8
Score for mfe	:	1.8
Score for randfold	:	1.6
Score for cons. seed	:	
Total read count	:	10
Mature read count	:	9
Loop read count	:	0
Star read count	:	1

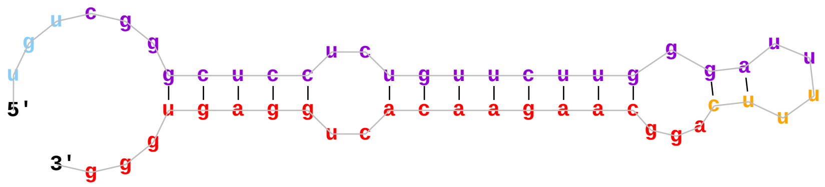

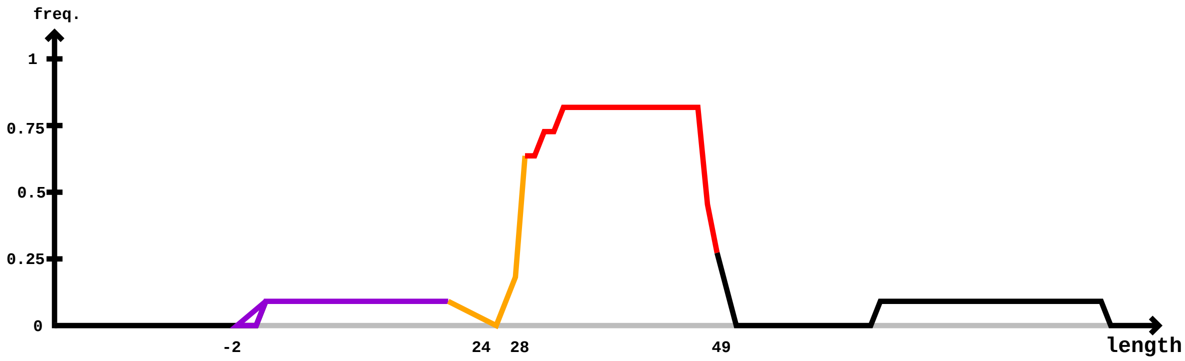

Star Mature

```
5'- ccccauggacuguagcuugucgggcuccucuguucuugggauuuuucaggcaagaacacuggaguggguugcaaccauuuccuccuccaggggaucuucccgacccagggaua  -3'   obs
    ccccauggacuguagcuugucgggcuccucuguucuugggauuuuucaggcaagaacacuggaguggguugcaaccauuuccuccuccaggggaucuucccgacccagggaua         exp
    ((((.((((.((((((((.....(((((..((((((((.((....))...))))))))..)))))))))))))............))))))))....((((......))))..   reads   mm   sample
    ....................cgggcuccucuguucuugggauu......................................................................   1       0    seq
    .............................................ucaggcaagaaUacuggaguggg.............................................   1       1    seq
    ..............................................caggcaagaacacuggagugg..............................................   1       0    seq
    ...............................................aggcaagaacacuggaguU...............................................   1       1    seq
    ...............................................aggAaagaacacuggagug...............................................   1       1    seq
    ...............................................aggcaagaacacuggagGg...............................................   1       1    seq
    ...............................................aggcaagaacacuggagugg..............................................   1       0    seq
    ...............................................Cggcaagaacacuggaguggg.............................................   1       1    seq
    .................................................gcaagaacacuggaguA...............................................   1       1    seq
    ...................................................aagaacGcuggagugggu............................................   1       1    seq
    ....................................................................................cuccaggggaucuucccgacccaC.....   1       1    seq
```
